# Proposal of a diagnostic algorithm for myofascial trigger points based on a multiple correspondence analysis of cross-sectional data

**DOI:** 10.1186/s12891-023-06129-y

**Published:** 2023-01-24

**Authors:** Petra Baeumler, Kerstin Hupe, Dominik Irnich

**Affiliations:** Multidisciplinary Pain Center, Department of Anaesthesiology, University Hospital LMU, 80336, Pettenkoferstr 8a, Munich, Germany

**Keywords:** Myofascial pain syndrome, Taut band, Referred pain, Nodule, Pressure pain, Hypersensitive spot, Local twitch response, Muscular dysfunction, Diagnostic criteria, Multiple correspondence analysis

## Abstract

**Background:**

Myofascial trigger points (MTrPS), the morphological correlate of myfascial pain syndromes (MPS), contribute to the worldwide high chronic pain burden. However, uncertainty about MTrP diagnostic criteria remains. Aim of this cross-sectional study was to characterize clusters of diagnostic criteria assessable during physical examination that might guide MTrP diagnosis.

**Methods:**

Thirteen MTrP diagnostic criteria proposed in relevant literature were assessed by standardized examinations in the trapezius and levator scapulae muscles of 61 chronic pain patients undergoing an interdisciplinary pain assessment. Hierarchical cluster analysis from multiple correspondence analysis was applied to data of the four muscles separately. Examining physicians classified the findings as MTrP, sufficient for diagnosis of an MPS and/or relevant for the patients’ pain condition.

**Results:**

Taut bands, hypersensitive spots within a taut band, nodules within a taut band and referred pain (classical diagnostic criteria) were most frequent (28–66% M. trapezius, 8–21% M. levator scapulae). Restricted range of motion, pain during contraction, pain exacerbation during emotional stress, muscular weakness, jump sign, local twitch response and autonomic phenomena (complementary diagnostic criteria) occurred in 2–25% and hypersensitive spots and nodules outside of a taut band in 2–7% of the cases. Four clusters emerged: (1) no or just one diagnostic criterion, mostly a taut band alone; (2) a hypersensitive spot and/or nodule outside of a taut band partly in combination with complementary diagnostic criteria; (3) at least two classical diagnostic criteria (mostly a taut band containing a hypersensitive spot) partly in combination with complementary diagnostic criteria; (4) at least two, rather three, classical diagnostic criteria always in combination with complementary diagnostic criteria. Referred pain was specific to cluster 3 and 4. Among classical diagnostic criteria, palpable nodules within a taut band contributed least, and among complementary diagnostic criteria, restricted range of motion and pain during contraction contributed most to data representation.

**Conclusion:**

We propose that the definite diagnosis of an MTrP requires a hypersensitive spot potentially felt as a nodule located within a taut band in addition to either referred pain, a local twitch response or at least two complementary diagnostic criteria, whereby signs of muscular dysfunction take on greater importance.

**Supplementary Information:**

The online version contains supplementary material available at 10.1186/s12891-023-06129-y.

## Background

Myofascial trigger points (MTrPs) are understood as the morphological correlate of the myofascial pain syndrome (MPS), an acute or chronic muscular pain condition affecting a single muscle or a group of muscles. Active MTrPs are spontaneously painful, while latent MTrPs are only painful upon pressure. MTrP stimulation can also cause referred pain and the characteristic local twitch response [1–3].

The etiology of MTrPs is still incompletely understood. The so called integrated trigger point hypothesis summarizes observations of dysfunctional motor endplates along with incessant fiber contraction and a consequent energy crisis causing local ischemia and hypoxia with increasing concentrations of vasoactive, neuromodulatory and pro-inflammatory substances. Continuous activation and sensitization of primary muscle nociceptors can contribute to central sensitization processes and pain chronification. The development of MPS, as any other chronic pain condition, needs to be understood as a multifactorial process within the framework of the bio-psycho-social model. (see [4, 5] for review).

MTrPs are thought to contribute not only to musculoskeletal disorders, such as neck pain, back pain, whiplash associated disorder [6], osteoarthritis [7, 8] and temporomandibular disorder [9, 10], but also to migraine and tension-type headache [11], pelvic pain [12] and even cancer pain [13]. These, often chronic pain conditions, in particular musculoskeletal disorders, are highly prevalent and cause substantial disease burden [14] and socioeconomic costs [15, 16].

The most extensive work on MTrPs owes to Travell and Simons. Originally they defined an MTrP as “… a hyperirritable spot, usually within a taut band of skeletal muscle or in the muscle’s fascia. The spot is painful on compression and can give rise to characteristic referred pain, tenderness, and autonomic phenomena.” [17]. Subsequently, electrophysiological and histological insights [18–22] prompted a more specific definition provided in the second edition of Simons’ and Travell’s standard textbook: “A hyperirritable spot in skeletal muscle that is associated with a hypersensitive palpable nodule in a taut band. …” [3]. Here, it was also recognized that an MTrP can also cause motor dysfunction, such as muscular weakness or reduced muscle elongation. In a later paper Simons argued with reference to work by Gerwin and Dommerholt that the minimal set of diagnostic criteria for a latent MTrP were a hypersensitive spot within a taut band whose palpation caused referred pain [23].

This historical evolvement lead to controversies and varying definitions of necessary, sufficient and complementary MTrP diagnostic criteria within standard text books [1, 2] and the scientific literature on MTrP diagnostic reliability and prevalence [6, 24, 25]. Pain experts seem to disaccord particularly on the relevance of palpable nodules and referred pain as well as complementary signs and symptoms [26, 27]. The latest consensus on MTrP diagnostic criteria attempted in a Delphi study resulted in a minimal set of two out of three criteria—taut band, hypersensitive spot and referred pain [28]. However, the discussion about palpable nodules was omitted, and experts considering a referred sensation an essential MTrP diagnostic criterion were on par with those opposing this proposition.

Given the potentially important role of MTrPs in the pathogenesis of pain conditions, it can be inferred that proper diagnosis and treatment of MTrPs can mitigate their detrimental consequences. Thus, the aim of this study was to explore clusters of MTrP diagnostic criteria obtained during physical examination that might guide clinical diagnosis of MTrP and MPS in the future.

## Methods

### Study design

In this prospective cross-sectional study chronic pain patients undergoing an interdisciplinary assessment at the Multidisciplinary Pain Center, Department of Anaesthesiology, Campus City Center, University Hospital LMU Munich were consecutively included between August and October 2017. During physical examinations of the trapezius and the levator scapulae muscles (M. trapezius, M. levator scapulae) on both body sides, physician’s experienced in manual examination assessed thirteen MTrP diagnostic criteria and evaluated whether the particular finding was sufficient for the diagnosis of an MTrP, sufficient for the diagnosis for an MPS and/or relevant for the patient’s pain condition. Associations between MTrP diagnostic criteria and resulting patient clusters were evaluated by multiple correspondence analysis (MCA) with agglomerative hierarchical cluster analysis.

### Study population

Eligible were chronic pain patients 18 years or older undergoing an interdisciplinary pain assessment at the study center performed by two medical doctors (anesthetist / physical medicine doctor) and a psychologist. Patients can be referred to such interdisciplinary pain assessment (tertiary care service) either by their primary or secondary care physician or can arrange appointments themselves. Pain assessments also include consideration of previous diagnoses and laboratory test results as well as the eventual initiation of further imaging or laboratory diagnostics. An interdisciplinary pain assessment is a prerequisite for long-term comprehensive pain management service at the study center e.g. participation in a multidisciplinary pain program. Included patients suffered from either nociceptive, neuropathic or nociplastic pain disorders. Patients with acute pain were not included. Further exclusion criteria were conditions that impaired the patient’s reasoning capability, such as psychosis, intoxication, dementia or delirium. Sixty-one out of 62 eligible patients consented to participate in the study. One patient was below the age of 18 and was not included.

### Data collection

Eleven MTrP diagnostic criteria that can be feasibly assessed in a singular physical examination were identified from relevant literature [3, 6, 17, 18, 23, 25, 26, 28–30]: taut band, palpable nodule, hypersensitive spot, referred pain, jump sign, local twitch response, muscular weakness, restricted range of motion, pain during contraction, autonomic phenomena and pain exacerbation during emotional stress. Nodules and hypersensitive spots were categorized further according to their location within or outside of a taut band. Thus, 13 MTrP diagnostic criteria were assessed in total in this study. Diagnostic criteria referring to symptom reduction after treatment (injection of local anesthetics, dry needling or acupuncture) were not assessed. Given their supposed role in MPS diagnosis, recognition of local pain upon pressure or recognition of referred pain were also documented.

**Fig. 1:**
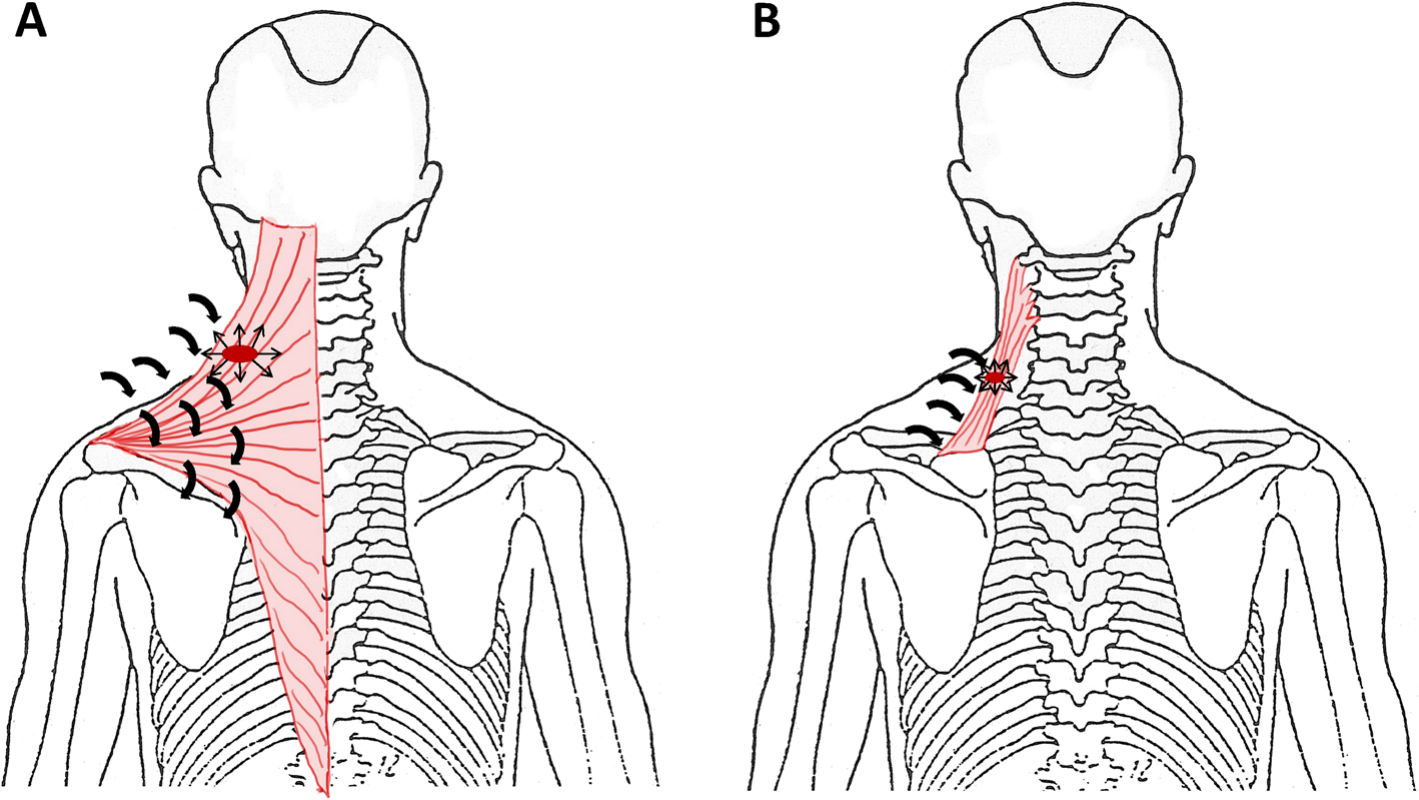
Examination of the M. trapezius (**A**) and M. levator scapulae (**B**) by palpation. Arrows indicate the direction of the palpation perpendicular to the muscle structure

MTrP diagnostic criteria were assessed each in the left and right M. trapezius and M. levator scapulae during physical examinations standardized according to standard text books [1, 31]. The five participating physicians received detailed instructions by the principal investigator, including explanatory notes on the MTrP diagnostic criteria to be assessed. First, physicians inspected the patient’s posture, anatomy and the area over the respective muscles to detect aberrations in muscle tone and autonomic phenomena. Second, the examiner tested whether the mobility of the cervical spine (active and passive rotation, inclination, flexion, extension, rotation in flexion and in extension) was restricted or painful. Third, the trapezius (Fig. 1A) and levator scapulae muscles (Fig. 1B) were palpated perpendicularly to the fiber structure.

In case of any anomaly, such as a nodule, a taut band, local or referred pain upon pressure, autonomic phenomena or others, the respective area was examined in closer detail by inspection and further palpation. Elicitation of local and referred pain was attempted by pressure application through perpendicular pressing or pincer grip maintained for several seconds. For each of the four muscles, an independent observer (KH) documented the location of the first prominent myofascial finding and ticket each of the MTrP diagnostic criteria (Table 1) with either 1 = „yes criterion present “ or 0 = „no—criterion not present “ in the case report form.

**Table 1:** Patient and physician characteristic and frequencies of diagnostic criteria

**Patient characteristics**					
**Age [a]**, m ± SD (min – max)		51.0 ± 15.1 (21 – 84)			
**BMI [kg/m**^**2**^**]**, m ± SD (min – max)		25.1 ± 5.1 (13 – 43)			
**Gender**, n (%)	**female / male**	40 (66) / 21 (34)			
**MPSS**, n (%)	**stage 1**	1 (2)			
	**stage 2**	17 (28)			
	**stage 3**	43 (70)			
**Number of diagnoses**, m ± SD (min – max)		3.7 ± 1.5) (1 – 8)			
**Physician characteristics**					
**Age [a]**, m ± SD (min – max)		44.2 ± 9.3 (32—54)			
**Gender**, n (%)	**female / male**	- / 5 (100)			
**Clinical experience**		15.0 8.3 (4 – 24)			
** Medical specialization**	**Physical and rehabilitation medicine**	4 (80)			
	**Anesthesia**	1 (20)			
** Additional qualifications**	**Pain medicine**	1 (20)			
	**Manual medicine**	5 (100)			
	**Sports medicine**	3 (60)			
	**Acupuncture**	2 (40)			
**Diagnostic criteria**, n (%)		**M. trapezius**		**M. levator scapulae**	
		**left**	**right**	**left**	**right**
Taut band		38 (62)	40 (66)	11 (18)	13 (21)
Nodule	within a taut band	19 (31)	17 (28)	8 (13)	3 (5)
	outside of a taut band	2 (3)	-	-	-
Spot hypersensitive to pressure	within a taut band	30 (49)	28 (46)	10 (16)	8 (13)
	of those with recognizable local pain	22 (36)	25 (41)	8 (13)	5 (8)
	outside of a taut band	4 (7)	4 (7)	1 (2)	3 (5)
	of those with recognizable local pain	1 (2)	1 (2)	1 (2)	1 (2)
Referred pain	total	18 (30)	20 (33)	7 (11)	5 (8)
	of those recognizable referred pain	13 (21)	15 (25)	5 (8)	5 (8)
Jump sign		7 (11)	6 (10)	1 (2)	2 (3)
Local twitch response		1 (2)	-	-	-
Restricted range of motion		10 (16)	7 (11)	3 (5)	5 (8)
Muscular weakness		6 (10)	2 (3)	2 (3)	3 (5)
Pain during contraction		14 (23)	9 (15)	3 (5)	4 (7)
Autonomic phenomena		2 (3)	2 (3)	-	-
Pain exacerbation during emotional stress		15 (25)	11 (18)	5 (8)	5 (8)

*M* mean, *SD* standard deviation, *min* minimum, *max* maximum, *n* (%) absolute and relative frequency, *a* years, *kg* kilogram, *m* meter, *MPSS* Mainz Pain Staging System, *M* musculus

Finally, examining physicians clinically evaluated whether the respective finding was sufficient for the diagnosis of an MTrP and/or an MPS (1 = “yes”, 0 = “no”) and whether it contributed relevantly to the patient’s pain condition. Physicians filled a questionnaire about their medical specialty, additional training and years of clinical experience in physical examination. Patient characteristics (age, sex, height, weight, BMI, pain diagnosis and the stage of pain chronification according to the Mainz pain staging system (MPSS) [32]) were extracted from medical records.

### Data analyses

Data analyses were carried out with the statistical soft wares SPSS version 24 [33] and R version 3.5.1 [34]. Continuous variables are represented as means ± standard deviations as well as value ranges and categorical variables as absolute and relative frequencies.

Associations between MTrP diagnostic criteria were visualized by MCA. MCA allows data representation on a reduced number of orthogonal dimensions that optimally separate the categories of the variables (yes and no) and cases with different combinations of variable categories while preserving the diversity of combinations of categories as much as possible [35]. The number of dimensions retained was determined according to the Kaiser criterion [36]. In MCA plots, variable categories (diagnostic criteria present or absent) were color-coded based on their contributions to the dimension. Based on MCA results, hierarchical cluster analysis was used to determine clusters of patient cases according to concomitantly occurring MTrP diagnostic criteria. Associations between the dimensions and the general patient characteristics as well as the physicians’ clinical evaluations were assessed by correlation analyses.

## Results

### Patient and physician characteristics

Characteristics of the 61 included patients and physicians as well as frequencies of identified diagnostic criteria are depicted in Table 1.

MTrP diagnostic criteria in at least one of the four muscles were identified in 55 patients (90%). Diagnostic criteria were more frequent in the trapezius than the levator scapulae muscles. Most frequently identified criteria were taut bands, hypersensitive spots within taut bands followed by palpable nodules within taut bands and referred pain. In the following these are referred to as classical MTrP diagnostic criteria in line with the literature outlined above. Two thirds of the trapezius muscles and one third of the levator scapulae muscles exhibited a taut band of which over two thirds contained a hypersensitive spot and/or a palpable nodule either with or without referred pain. The remaining criteria—jump sign, local twitch response, restricted range of motion, muscular weakness, pain during contraction, autonomic phenomena and pain exacerbation during emotional stress—occurred less frequently and are referred to as complementary diagnostic criteria. These occurred almost exclusively in combination with classical diagnostic criteria or hypersensitive spots or palpable nodules outside of a taut band that were identified in only 2–7% of the cases. Jump signs were only elicited in the presence of a hypersensitive spot within a taut band.

### Clusters of MTrP diagnostic criteria

**Fig. 2:**
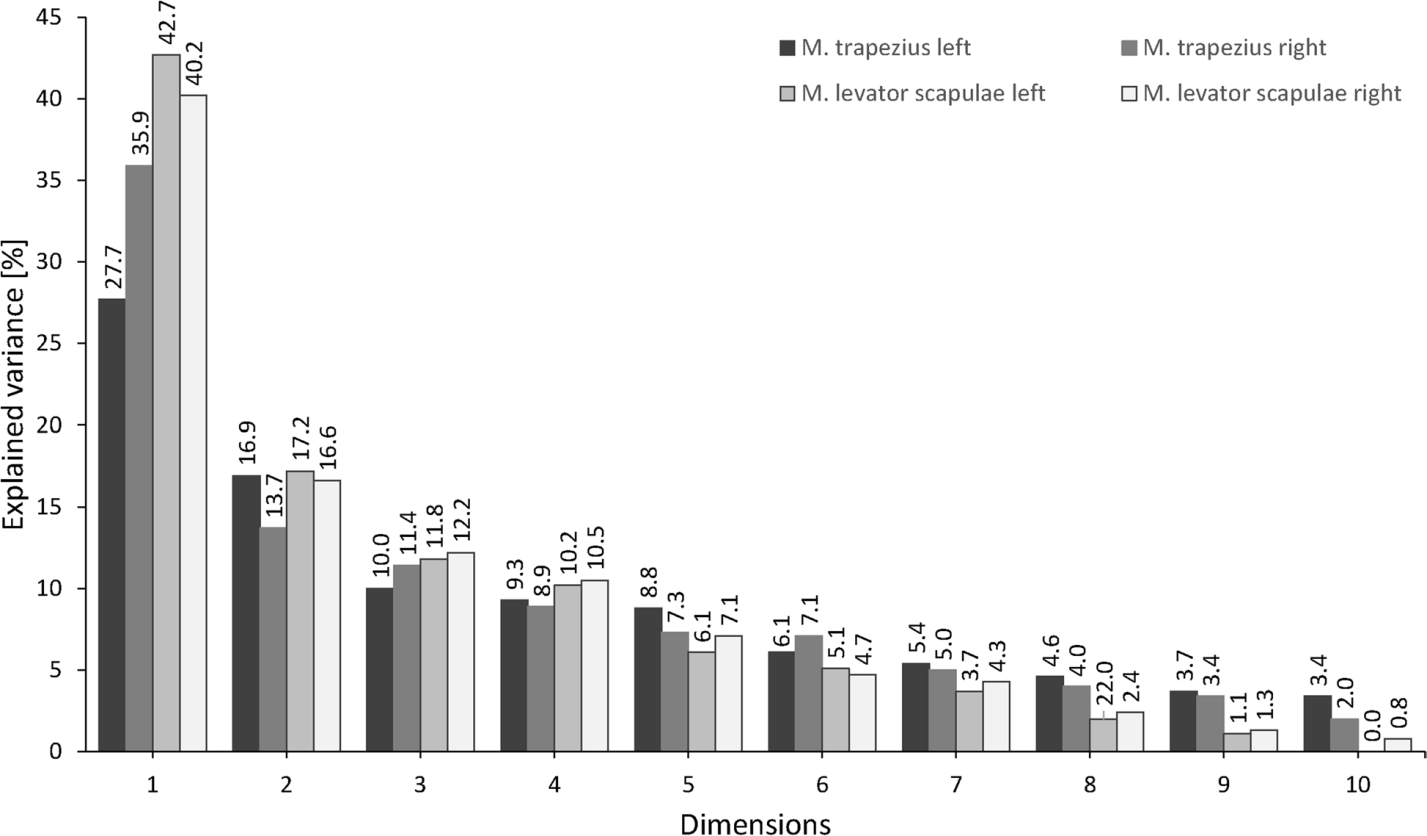
Scree plot

The empirical Kaiser Criterion indicated a two dimensional solution by the MCA explaining 44.6% – 59.9% of the variance in the data of the four muscles (Fig. 2).

**Fig. 3:**
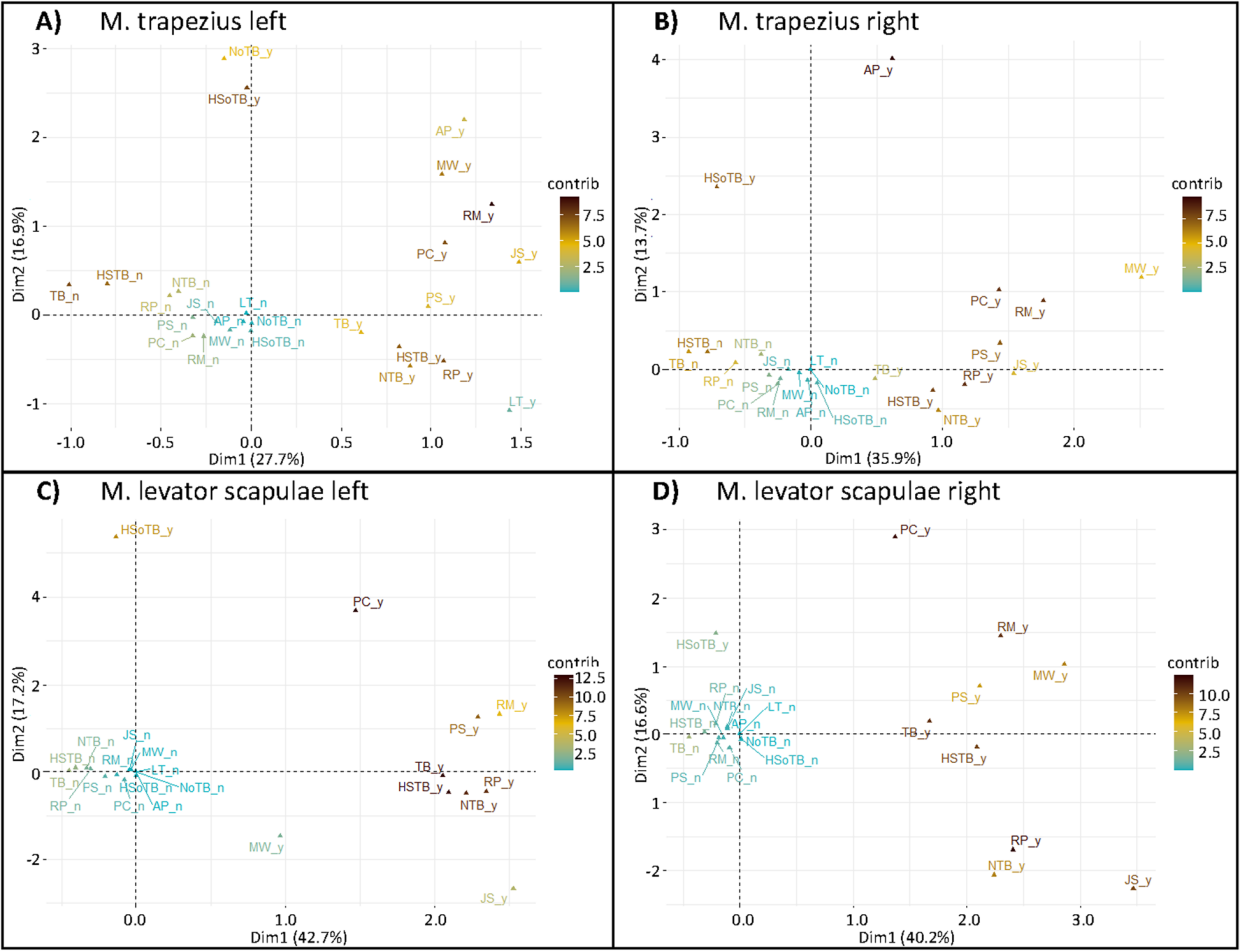
MCA-plots of categories of MTrP diagnostic criteria. M. trapezius left (**A**) M. trapezius right (**B**) M. levator scapulae left (**C**) M. levator scapulae right (**D**); TB: taut band; NTB: nodule within a taut band; NoTB: nodule outside of a taut band; HSTB: hypersensitve spot within a taut band; HSoTB: hypersensitive spot outside of a taut band; RP: referred pain; JS: jump sign, LT: local twitch response; RM: restricted range of motion; MW: muscular weakness; PC: pain during contraction; AP: autonomic phenomena; PS: pain exacerbation during emotional stress; _y: yes – diagnostic criterion present, _n: no – diagnostic criterion not present; Dim1: dimension 1; Dim2: dimension 2

MCA plots (Fig. 3A-D) represent the yes- and the no-category of each MTrP diagnostic criterion colored according to their contribution. Accumulation of the no-categories near the origin reflects the substantial proportion of individuals without any diagnostic criterion. Positive scores on dimension 1 represent combinations of classical MTrP diagnostic criteria which are located near to each other in line with their mutual association. Classical diagnostic criteria contributing most to data representation were hypersensitive spots within taut bands (yes), referred pain (yes) and taut bands (no – trapezius muscles, yes – levator scapulae muscles). Dimension 2 quantified the presence of complementary diagnostic criteria and hypersensitive spots or nodules outside of a taut band which are located furthest from the origin reflecting their rarer occurrence. Positive scores on dimension 1 of complementary diagnostic criteria reflect their association with classical diagnostic criteria. Pain during contraction (yes) and/or restricted range of motion (yes) were complementary diagnostic criteria with the largest overall contributions in all four muscles. Yes categories of hypersensitive spots and nodules outside of a taut band contributed to dimension 2 only.

**Fig. 4:**
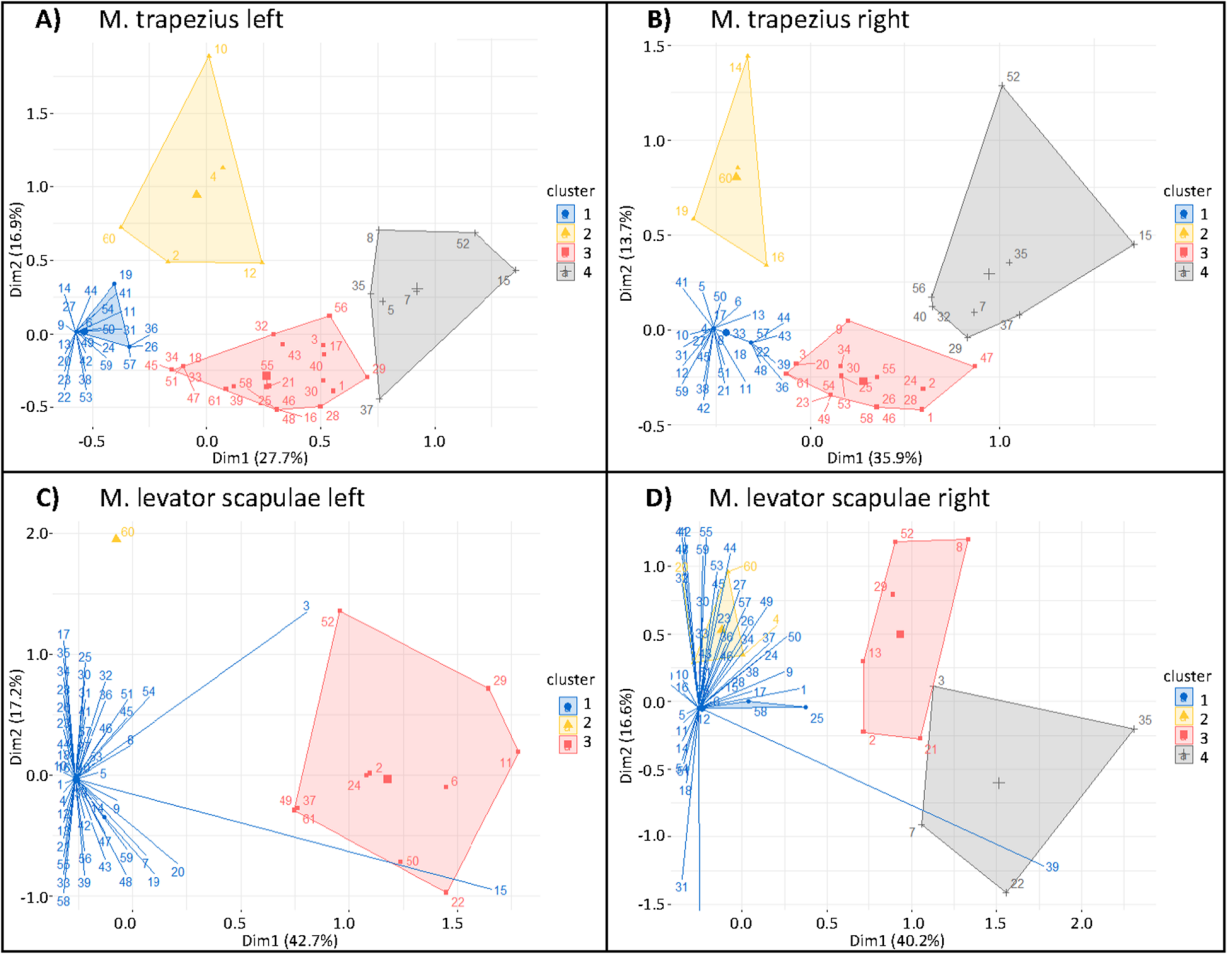
MCA plot of point clouds with hierarchical cluster analysis. Four cluster solution for the M. trapezius left (**A**), the M. trapezius right (**B**) and the M. levator scapulae right (**D**); three cluster solution for the M. levator scapulae (**C**) due to the limited number of identified complementary diagnostic criteria. (The four cluster solution subsumed two individuals with opposite coordinates on dimension 1, because they shared the rare yes-category for muscular weakness.) Dim1: dimension 1; Dim2 = dimension 2

Hierarchical cluster analysis based on MCA results provided a four-cluster solution for the left and right M. trapezius and the right M. levator scapulae (Fig. 4 A, B, D) and a three cluster solution for the M. levator scapulae (Fig. 4 C). The four clusters in the four muscles resembled each other and were characterized as follows (Table 2):

**Table 2:** Frequency of diagnostic criteria and clinical evaluation within clusters derived from the MCA

				**Cluster 1**				**Cluster 2**				**Cluster 3**				**Cluster 4**			
				**M. trapezius**		**M. lev. scap**		**M. trapezius**		**M. lev. scap**		**M. trapezius**		**M. lev. scap**		**M. trapezius**		**M. lev. scap**	
				**left**	**right**	**left**	**right**	**left**	**right**	**left**	**right**	**left**	**right**	**left**	**right**	**left**	**right**	**left**	**right**
				*n* = 24	*n* = 28	*n* = 49	*n* = 48	*n* = 5	*n* = 4	*n* = 1	*n* = 3	*n* = 25	*n* = 20	*n* = 11	*n* = 4	*n* = 7	*n* = 9	-	*n* = 6
**Combinations of classical MTrP diagnostic criteria** (mutually exclusive) n (%)																			
** None**				21 (88)	19 (68)	49 (100)	46 (96)	2 (40)	2 (50)	1 (100)	2 (67)	-	-	-	-	-	-	na	-
**TB**				3 (13)	9 (32)	-	1 (2)	2 (40)	1 (25)	-	1 (33)	-	-	1 (9)		-	-	na	1 (17)
**TB**	**HSTB**			-	-	-	1 (2)	-	-	-	-	8 (32)	4 (20)	-	-	1 (14)	1 (11)	na	3 (50)
**TB**		**NTB**		-	-	-	-	1 (20)	1 (25)	-	-	1 (4)	1 (5)	-	1 (25)	-	-	na	-
**TB**			**RP**	-	-	-	-	-	-	-	-	-	-	-	-	-	-	na	1 (17)
**TB**	**HSTB**	**NTB**		-	-	-	-	-	-	-	-	3 (12)	3 (15)	3 (27)	-	1 (14)	-	na	-
**TB**	**HSTB**		**RP**	-	-	-	-	-	-	-	-	3 (12)	4 (20)	2 (18)	1 (25)	2 (29)	4 (44)	na	1 (17)
**TB**		**NTB**	**RP**	-	-	-	-	-	-	-	-	1 (4)	-	-	-	-	-	na	-
**TB**	**HSTB**	**NTB**	**RP**	-	-	-	-	-	-	-	-	9 (36)	8 (40)	5 (45)	2 (50)	3 (43)	4 (44)	na	-
**Hypersensitive spot / nodule outside of a taut band** (multiple answers possible) n (%)																			
** HSoTB**				-	-	-	-	4 (80)	4 (100)	1 (100)	3 (100)	-	-	-	-	-	-	na	-
** NoTB**				-	-	-	-	2 (40)				-	-	-	-	-	-	na	-
**Complementary diagnostic criteria** (multiple answers possible) n (%)																			
** Jump sing**				-	-	-	-	-	-	-	-	3 (12)	3 (15)	1 (9)	2 (50)	4 (57)	3 (33)	na	-
** Local twitch response**				-	-	-	-	-	-	-	-	-	-	-	-	1 (14)	-	na	-
** Restricted range of motion**				-	-	-	-	2 (40)	-	-	-	1 (4)	2 (10)	3 (27)	2 (50)	7 (100)	5 (56)	na	3 (50)
** Muscular weakness**				1 (4)	-	1 (2)	-	1 (20)	-	-	-	-	-	1 (9)	2 (50)	4 (57)	2 (22)	na	1 (17)
** Pain during contraction**				-	-	-	-	3 (60)	1 (25)	1 (100)	1 (33)	6 (24)	-	2 (18)	-	5 (71)	8 (88)	na	3 (50)
** Autonomic phenomena**				-	-	-	-	1 (20)	1 (25)	-	-	-	-	-	-	1 (14)	1 (11)	na	-
** Pain exacerbation emot. stress**				-	-	-	-	2 (40)	-	-	-	10 (40)	4 (20)	5 (45)	1 (25)	3 (43)	7 (78)	na	4 (67)
**Any complementary diagnostic criterion** n (%)																			
				1 (4)	-	1 (2)	-	5 (100)	2 (50)	1 (100)	1 (33)	12 (48)	8 (40)	8 (73)	3 (75)	7 (100)	9 (100)	na	6 (100)
**Total number of complementary diagnostic criteria** (min – max)																			
				(0—1)	-	(0—1)	-	(1—3)	(0—1)	1	(0—1)	(0—3)	(0—2)	(0—3)	(0—4)	(2—5)	(2—5)	na	(1—3)
**Clinical evaluation** n (%)																			
** Myofascial trigger point**							2 (4)	2 (40)				19 (76)	15 (75)	11 (100)	3 (75)	7 (100)	9 (100)	na	5 (83)
** Myofascial pain syndrome**								1 (20)				15 (60)	13 (65)	10 (91)	3 (75)	6 (86)	9 (100)	na	4 (67)
** Relevance for clinical pain**								3 (60)	1 (25)	1 (100)	1 (33)	17 (68)	15 (75)	10 (91)	3 (75)	5 (71)	9 (100)	na	4 (67)

*TB* taut band, *NTB* nodule within a taut band, *NoTB* nodule outside of a taut band, *HSTB* hypersensitive spot within a taut band, *HSoTB* hypersensitive spot outside of a taut band, *RP* referred pain, *na* not applicable

- **Cluster 1:** No or only one MTrP diagnostic criterion, mostly a taut band alone.
- **Cluster 2:** Hypersensitive spot and/or a nodule outside of a taut band commonly in combination with complementary diagnostic criteria.
- **Cluster 3:** Combination of at least two classic diagnostic criteria in the absence of or in combination with few (one to four) complementary diagnostic criteria.
- **Cluster 4:** Combination of at least two (rather three) classic diagnostic criteria always in combination with complementary diagnostic criteria (one to five).

The classical diagnostic criteria in cluster 3 and 4 were mostly a hypersensitive spot within a taut band either in combination with or in the absence of referred pain and a palpable nodule. Referred pain was limited to cluster 3 and 4. Complementary diagnostic criteria differentiating cluster 4 from cluster 3 were those related to muscular dysfunction; restricted range of motion and pain during contraction followed by muscular weakness. Pain exacerbation during emotional stress was strongly associated with classical diagnostic criteria (located closer in MCA plots) and occurred in considerable proportions of cluster 3 and 4. The few cases of autonomic phenomena appeared in cluster 2 and 4 cases. The single case with a local twitch response exhibited all classical diagnostic criteria.

### Association of patient characteristics and examiners with diagnostic criteria

Patient characteristics were neither associated with scores on dimension 1 nor with scores on dimension 2. The examiner ID was weakly associated with dimension 2 (*r* = 0.293 *p* < 0.01) in the left M. trapezius. This related to one examiner who identified complementary diagnostic criteria in more patients than the other examiners (83% vs 50 – 65%).

### Association of clinical evaluations with clusters of diagnostic criteria

Scores on dimension 1 were closely associated with the clinical diagnoses of an MTrP (*r* 0.6 – 0.9 *p* ≤ 0.01) and an MPS (*r* 0.6 – 0.9 *p* ≤ 0.01) as well with the appraisal of the finding as relevant for the patient’s pain condition (*r* 0.5 – 0.7 *p* ≤ 0.01). Scores on dimension 2 were not associated with clinical evaluations. An MTrP and an MPS was diagnosed in nearly all cluster 4 cases and in the vast majority of cluster 3 cases, but only in single cluster 1 and 2 cases (Table 2, ${\mathrm{\rm X}}^{2}$-Test *p* < 0.001). Cluster 3 and 4 cases were also rated more frequently as relevant for the pain condition (Table 2, ${\mathrm{\rm X}}^{2}$-Test *p* < 0.001). An MTrP was diagnosed in all cases with a hypersensitive spot within a taut band plus referred pain.

**Fig. 5:**
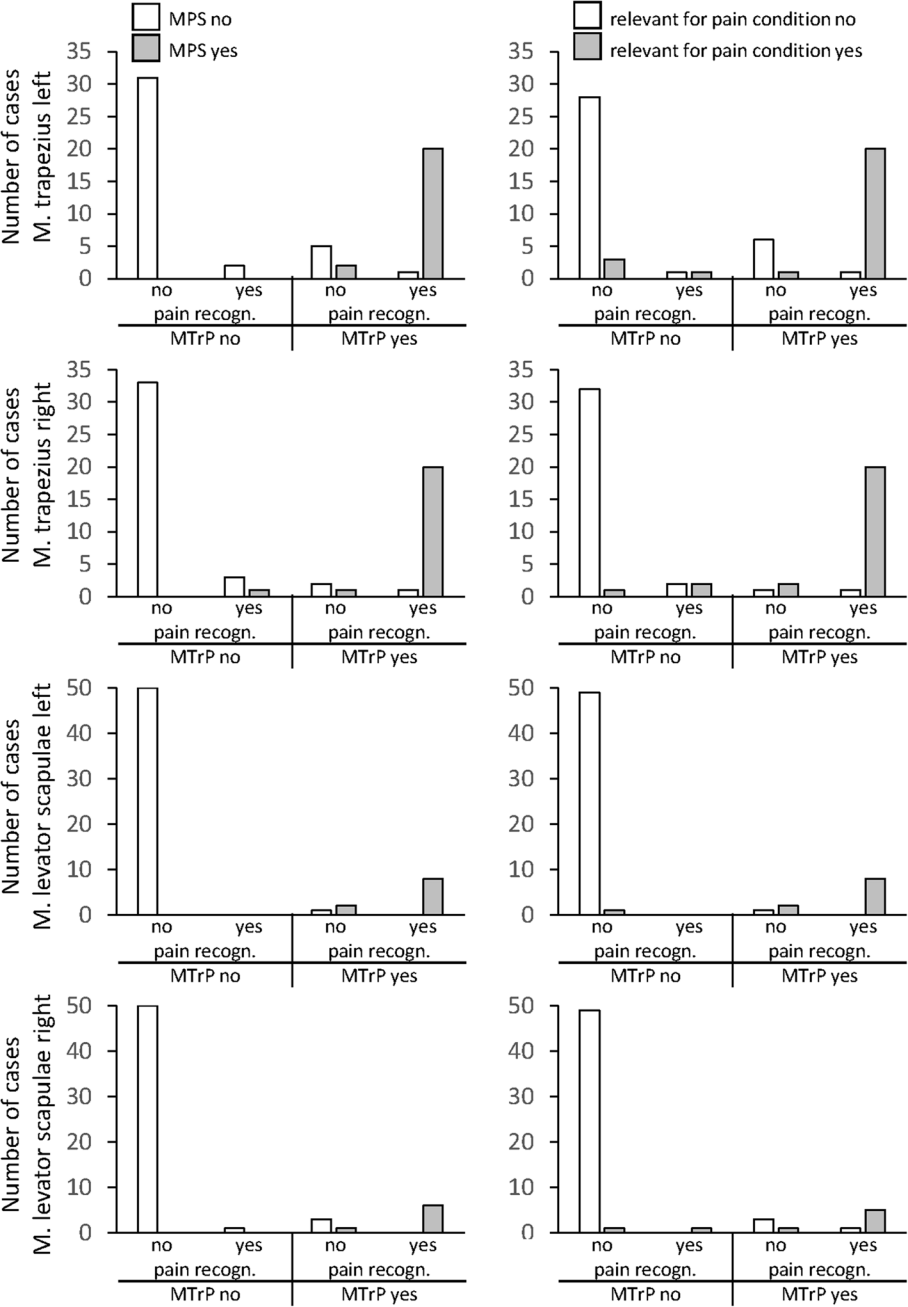
Associations between clinical evaluations and pain recognition. MTrP: myofascial trigger point; MPS: myofascial pain syndrome; pain recognition refers to recognized local pain upon pressure and/or recognized referred pain

Classification as an MTrP led in large part to the diagnosis of an MPS (M. trapezius left 79% and right 88%, M. levator scapulae left 91% and right 70%) and to assuming relevance for the patient’s pain condition (M. trapezius left 75% and right 91%, M. levator scapulae left 91% and right 60%). In addition to an identified MTrP, the diagnosis of an MPS was based mostly on the recognition of either local or referred pain. In single cases diagnosed as MPS, neither local nor referred pain was recognized by the patient (Fig. 5).

## Discussion

### Main findings

This is the first study evaluating clustering of MTrP diagnostic criteria identifiable by manual physical examination to assess their relevance.

Our findings provide a purely data driven justification and modification for the proposed expert consensus on MTrP diagnosis [28]. Concordantly, taut band, hypersensitive spot and referred pain were identified as the most essential MTrP diagnostic criteria, but importantly and in line with the definition by Travell and Simons [31], our results suggest that a hypersensitive spot needs to be located within a taut band, and that the simultaneous occurrence of referred pain and/or complementary diagnostic criteria are necessary for a definite MTrP diagnosis. Consequently, palpation of a taut band alone does lead to the diagnosis of an MTrP.

MCA clearly separated cases with at least two classical diagnostic criteria with either few (cluster 3) or many complementary diagnostic criteria (cluster 4) from cases without any diagnostic criterion or just a taut band (cluster 1) and cases with hypersensitive spots or nodules outside of a taut band (cluster 2). Accordingly, clinicians classified the majority of cluster 3 and all cluster 4 but few cluster 1 and 2 cases as MTrPs. A hypersensitive spot within a taut band was by far the most prominent finding in cluster 3 and 4, and referred pain was unexceptionally restricted to these clusters. This reflects the eminent role of these two criteria in MTrP diagnosis. Clinicians classified all cases exhibiting a hypersensitive spot within a taut band and referred pain as MTrPs.

Conversely, among classical diagnostic criteria, palpable nodules within taut bands contributed least to data representation. Nodules within a taut band alone constituted few cases in cluster 2 and 3. Thus, their role in MTrP diagnosis by purely manual examination seems rather subordinate or just confirmative which corresponds to a prevalent expert opinion [26, 28]. This goes without contradicting their proven presence in MTrPs [18], as nodules might be un-identifiable by palpation in muscles located in deeper tissue layers.

Hypersensitive spots and nodules outside of a taut band (cluster 2) can represent anomalies other than MTrPs and should prompt further diagnosis to identify potential significant causes, such as tumors or swollen lymph nodes. Cluster 2 cases did not exhibit referred pain but several complementary diagnostic criteria and were rarely classified as MTrP. Cluster 2 was the smallest, but emerged in all four muscles and was not particular to certain examiners.

Complementary diagnostic criteria generally should entail muscle examination, as they occurred almost exclusively in combination with classical diagnostic criteria and with hypersensitive spots or nodules outside of a taut band. Accumulation of complementary diagnostic criteria, in particular those of muscular dysfunction, separated cluster 3 from cluster 4 reflecting their important role for increasing MTrP diagnostic certainty. Restricted range of motion and pain during contraction might especially substantiate MTrP diagnosis, as they contributed substantially to MCA data representation and were the most frequent in cluster 4. Muscular weakness contributed less to representation of the data and was found also in the absence of other MTrP diagnostic criteria. Pain exacerbation during emotional stress was associated with MTrPs in our study population, but from a clinical perspective, it represents a general phenomenon in pain conditions [37]. Jump signs occurred only in few cases. Taking into account that it represents a strong reaction to palpation of a hypersensitive spot, it might not contribute substantially to MTrP diagnosis in line with Simons and Travell [31]. The rareness of a local twitch response and autonomic phenomena renders them least important in MTrP diagnosis. Nevertheless, there is agreement about the high specificity of the local twitch response for MTrP diagnosis [23]. In clinical practice, its elicitation during dry needing may also assist MTrP diagnosis ex juvantibus. However, interrater reliability of the local twitch response has been shown to be low in palpatory examinations [38].

### Clinical implications for MTrP diagnosis

**Fig. 6:**
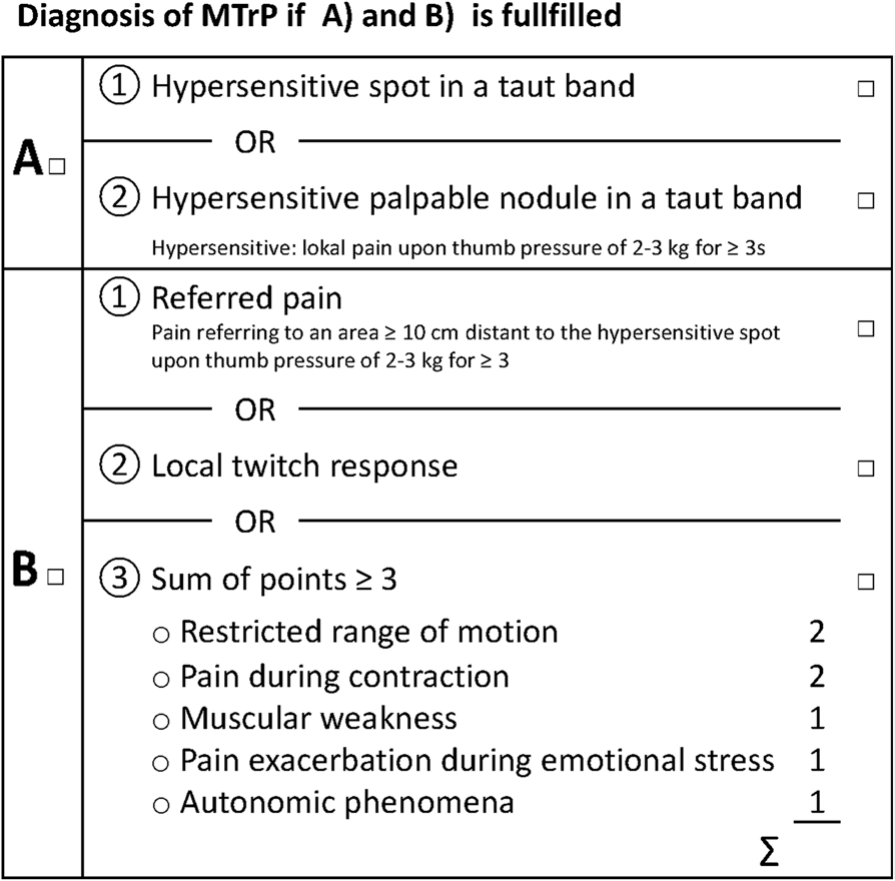
Proposed Munich Myofascial Trigger Point Score (MMTS)

Based on the considerations outlined above, we propose an MTrP diagnostic algorithm (Munich Myofascial Trigger Point Score, MMTS, Fig. 6). Identification of a taut band containing a hypersensitive spot potentially felt as a nodule appears most decisive for MTrP diagnosis which, according to our results, is only confirmed in combination with either referred pain, a local twitch response and/or at least two complementary diagnostic criteria (with restricted range of motion and pain during contraction taking on greater importance).

Reliability for the identification of hypersensitive spots within a taut band and referred pain appears good in comparison to other MTrP diagnostic criteria [24], but still varies largely between muscles and between studies, pointing to the need for examination standards. Elicitation of referred pain requires strong pressure stimulation, either perpendicularly to the taut band or by pincer grip, for several seconds. Pain can refer to loco-regional or distant sites. The referred pain patterns follow typically but not necessarily those described by Simons and Travell [28].

Our findings apply in particular to superficial muscles that are easy to access, as clusters of MTrP diagnostic criteria emerged particularly clearly in the M. trapezius. Examiners should also be trained in assessing sensory, functional and autonomic signs and symptoms. Obtained findings need to be categorized diagnostically e.g. by ruling out differential diagnosis. Improving accuracy of MTrP diagnosis is a prerequisite for treatments that aim at resolving MTrPs, such as dry needling and MTrP injection techniques, for which promising evidence exists [4, 5].

### Diagnosis of MPS

There is consensus that the MTrP is the morphological correlate of an MPS. However MPS definitions vary in preciseness with regard to differential diagnosis e.g. confined pain region versus widespread pain and recognition of pain or other symptoms [1–3, 28]. In our study the diagnosis MPS was mainly based on an identified MTrP with recognition of local pain upon pressure and/or recognition of referred pain, but additional information about co-morbidities have been considered by physicians. It seems appropriate to differentiate alternative reasons for pressure pain in muscle (e.g. myositis) and generalized pathologies causing wide spread pain including pressure pain in soft tissues (e.g. fibromyalgia). Therefore future research aiming to define and standardize MPS diagnosis should address differential diagnosis to assure adequate pain treatment.

### Strengths & limitations

Unlike previous studies, we investigated clusters of MTrP diagnostic criteria resulting from an MCA without a priori implications on clinical interpretation. Similarity between clusters emerging in the four muscles support generalizability of our finding. Physical examinations were standardized to reflect procedures recommended in standard text books. Furthermore, the examined MTrP diagnostic criteria were based on relevant up-to-date literature. Results were documented by an independent observer. Despite these strengths, our study has limitations: First, it was conducted in a mixed sample of consecutive chronic pain patients undergoing an interdisciplinary pain assessment, and documentation of diagnostic criteria were restricted to the trapezius and levator scapulae muscles. Future research needs to evaluate generalizability of our results to other muscles, e.g. deeper muscles and muscles in different body regions, as well as to different populations. Second, the sample size was comparable to e.g. studies on reliability of MTrP diagnosis [24], but larger samples are needed to confirm the proposed diagnostic algorithm. Third, differences in skills and examination styles of physicians are general challenges in research about manual techniques. This bias was minimized by standardized examination instructions.

## Conclusion

Our findings suggest a hypersensitive spot potentially felt as a palpable nodule within a taut band is a necessary MTrP diagnostic criterion, and that a definite diagnosis of an MTrP is established if in addition either referred pain, a local twitch response and/or several complementary diagnostic criteria in particular those reflecting muscular dysfunction are identified.

## Supplementary Information

**Additional file 1.**

## Data Availability

The dataset supporting the conclusions of this article along with a corresponding variable list is appended to this article as Additional file 1 (MTrPdia_BaeumlerHupeIrnich_data.xlsx). In order to protect patients’ identity clinical diagnoses not relevant for the data analyses were excluded.

## Abbreviations

a: Years
AP: Autonomic phenomena
Dim1: Dimension 1
Dim2: Dimension 2
HSoTB: Hypersensitive spot outside of a taut band
HSTB: Hypersensitve spot within a taut band
JS: Jump sign
kg: Kilogram
LT: Local twitch response
M: Musculus
m: Mean / meter
max: Maximum
min: Minimum
MMTS: Munich Myofascial Trigger Point Score
MTrP: Myofascial trigger point
MPS: Myofascial pain syndrome
MPSS: Mainz Pain Staging System
MW: Muscular weakness
NoTB: Nodule outside of a taut band
NTB: Nodule within a taut band
PC: Pain during contraction
PS: Pain exacerbation during emotional stress
RM: Restricted range of motion
RP: Referred pain
SD: Standard deviation
TB: Taut band
_y: Yes – diagnostic criterion present
_n: No – diagnostic criterion not present
